# Gender Differences in the Relationship Between Financial Capability and Health in Later Life: Evidence From Hong Kong

**DOI:** 10.1093/geroni/igad072

**Published:** 2023-07-07

**Authors:** Yu-Chih Chen, Sicong Sun

**Affiliations:** Department of Social Work and Social Administration, The University of Hong Kong, Pokfulam, Hong Kong SAR, China; Social Policy Institute, Washington University in St. Louis, St. Louis, Missouri, USA; School of Social Welfare, The University of Kansas, Lawrence, Kansas, USA

**Keywords:** Financial capability, Gender differences, Social determinants of health, Well-being

## Abstract

**Background and Objectives:**

Financial capability, comprising financial literacy, access, and behavior, can influence an individual’s ability to effectively use financial resources, thus affecting their health and well-being. However, studies have predominantly focused on financial literacy and overlooked a more comprehensive measure of financial capability and its health impacts. Furthermore, although financial capability is shaped profoundly by gender, there is limited knowledge of the role of gender in these associations.

**Research Design and Methods:**

This study investigated how gender may moderate the links between financial capability and health. The study recruited 1,109 community-dwelling adults (aged 45+) in Hong Kong to take part in an online survey employing multivariate linear and logistic regression to examine the gender differences in the associations between financial capability and physical (perceived health and mobility limitations), mental (life satisfaction and depression), and financial (retirement worry and financial satisfaction) health.

**Results:**

The results showed that financial access and behavior had a more significant influence on health outcomes than financial literacy. Gender differences in financial capability were identified through simple slope analyses. Financial literacy was more important for men’s self-rated health and life satisfaction, whereas financial behavior was more critical for women. Additionally, although financial access was not related to retirement worry among men, it was significantly associated with lower retirement worry among women.

**Discussion and Implications:**

The findings suggest that gender-specific pathways to financial capability may lead to health disparities. Policies and programs to improve population health and well-being, particularly for women, should target financial literacy, strengthen financial inclusion, and encourage responsible financial behavior.

**Translational Significance:** Financial capability, including financial literacy, access, and behavior, represents an individual’s ability to utilize financial resources effectively to enhance their health and well-being. This study highlights the role of financial capability as a determinant of health in later life while also demonstrating that gendered pathways to financial capability contribute to health disparities between men and women. Programs that expand financial access and improve financial knowledge and skills may benefit later-life health outcomes. Policies and programs should consider addressing multidimensional financial capability and gender equity to ensure desirable financial and health outcomes and equal opportunities for improved well-being for all.

Financial resources are essential for maintaining the health and well-being of individuals and households (Link & Phelan, 1995; Sun et al., 2022). Emerging research has suggested that financial capability—the ability and opportunities to use financial resources effectively—is a crucial social determinant affecting health (Sun & Chen, 2022). As a result, policymakers and practitioners have explored financial capability as an intervention to increase resources and reduce inequality (Sherraden, 2013). However, the empirical examination of the relationship between financial capability and health outcomes remains scant. Pathways and mechanisms linking financial capability and health outcomes are understudied. Understanding how financial capability affects health could inform interventions and policy designs to improve population health and well-being.

## Conceptualizing Financial Capability

This study uses Sherraden’s (2013) financial capability framework, which integrates intrinsic ability and external opportunity for financial actions. The financial capability framework draws upon Amartya Sen’s foundational theory of capabilities. According to the capability theory, both internal abilities and external conditions influence people’s opportunities for a secure and fulfilling life (Nussbaum, 2011; Sen, 1993). Financial capability, as an extension of this approach, is defined as the combination of a person’s *ability* to act (knowledge and skills) and their *opportunity* to act (access to inclusive financial services, products, and institutions) to improve well-being (Sherraden, 2013). Financial capability also recognizes that social and economic structures play a crucial role in shaping financial socialization, education opportunities, and financial inclusion related to policies, services, and products.

Financial capability is conceptualized as a combination of financial literacy, financial access, and financial behavior (Sherraden, 2013). Financial literacy is the ability to use knowledge and skills to manage financial resources (Huston, 2010). It includes understanding financial concepts and the attitude or confidence in using knowledge to make decisions across various financial contexts (Fernandes et al., 2014; Huston, 2010). Although the literature generally supports using an objective measure of financial literacy (e.g., correctness on financial tests, see Fernandes et al., 2014; Goyal & Kumar, 2021), research has also identified perceived financial literacy (e.g., financial confidence) as valuable as objective financial literacy in determining financial behavior (Allgood & Walstad, 2016). Financial access refers to the availability of affordable, safe, mainstream financial products and services (Birkenmaier et al., 2019), usually measured by the types of financial products an individual possesses (Despard et al., 2020; Sun et al., 2022). However, not all individuals have equal access to mainstream financial products and services due to differences in knowledge, resources, or power (e.g., by age, gender, education, or race; see Sherraden et al., 2018). Lastly, financial behavior is an individual’s actions and decisions regarding their financial resources. It encompasses various behaviors, such as budgeting and planning, borrowing, saving, and investing, and ability and confidence in money management (Kaiser & Menkhoff, 2017; Kaiser et al., 2022; Xiao et al., 2015). Financial behavior is typically characterized by responsible and effective management of financial resources, including setting and achieving financial goals, tracking expenses regularly, finding solutions when financial challenges occur, and making informed decisions about financial products and services (Robson & Peetz, 2020; Xiao et al., 2015).

The financial capability framework is in its early stages of development, and researchers are currently working on empirical evidence to demonstrate its effectiveness and suggest potential refinement. The term “financial capability” has been used widely across disciplines. However, researchers lack consensus or justification regarding the appropriate indicators to measure financial capability. Many studies use financial behaviors (such as money management, planning, and budgeting) as financial capability (Robson & Peetz, 2020; Taylor et al., 2011; von Stumm et al., 2013), whereas others measure financial capability as financial literacy (Lamb, 2016) or financial access (Friedline & West, 2016). Some studies use different combinations of financial capability, such as literacy and behavior (Rothwell et al., 2016; Xiao & O’Neill, 2018) or literacy and access (Despard et al., 2020).

Birkenmaier et al. (2022) conducted a scoping review and focused on the measurement of consumers’ financial capability in the scholarly literature published between 2015 and 2018. They call for standardization and harmonization of measurement to allow for internal consistency of findings and replication among studies. In an effort to establish theoretical and empirical links between financial capability and population health, Sun and Chen (2022) modeled financial capability as one latent construct. They recognized the limitations of this approach and recommended that future studies test the nuances of the subcomponents of financial capability and their relation to health to find potential malleable policy and program levers to improve well-being and reduce inequality most effectively. Additionally, Sun et al. (2022) measured financial literacy and access, and behavior as separate constructs to examine which aspect of financial capability should be targeted to reduce economic hardship. They found that relative to financial literacy, financial access plays a more prominent role in shaping financial behavior and reducing household economic hardship among the general U.S. population. They emphasized the importance of expanding financial access and promoting financial inclusion to achieve financial well-being and development.

As discussed by Sun and Chen (2022), financial capability is multidimensional and context-specific. Following recommendations from recent advances in financial capability research, we test three key constructs of the financial capability framework—financial literacy, financial access, and financial behavior. By examining these components of financial capability separately and simultaneously, we aim to assess and compare which areas of financial capability offer opportunities for intervention in working with older adults in Hong Kong.

### Theorizing Financial Capability as Determinants of Health

Sherraden’s (2013) financial capability framework proposes a sequential process to demonstrate how structural systems may affect an individual’s financial outcomes. Social and economic structures, such as banking systems, credit policies, and financial practices, may influence financial socialization and availability of financial products, subsequently affecting financial literacy, access, and behaviors and ultimately leading to better financial development and stability. Although this framework does not specifically consider health outcomes, financial capability can be further theorized as a determinant of health by incorporating two interrelated frameworks: social determinants of health (Marmot et al., 2008) and the theory of fundamental causes (Link & Phelan, 1995).

Social determinants of health are defined as the structural factors that affect health and well-being in which people are born, live, work, and age (Marmot et al., 2008). Upstream social determinants—economic resources, social systems (e.g., education, occupation, or policy), or social norms (e.g., equality or inequality towards gender, race, and others)—are macro factors that fundamentally shape downstream health conditions, such as attitudes, beliefs, and behaviors toward medical care and health. The theory of fundamental causes of diseases suggests that the fundamental causes of health are access to resources linked to money, power, and privilege. Therefore, the social determinants of health and fundamental causes of diseases are interconnected, as social determinants are the fundamental drivers of the conditions of economic resources and access to goods, education, services, and health care closely associated with people’s living environment, occupation, and status attainment over the lifespan (Braveman & Gottlieb, 2014). These play a significant role in determining a person’s access to resources and, in turn, affect health outcomes later in life (Marmot et al., 2008).

In this vein, financial capability is considered a determinant of health because financial capability represents an individual’s ability and opportunities to act (Sun & Chen, 2022). People with higher levels of financial capability—having greater access to financial services and products, being equipped with improved financial knowledge and skills, or demonstrating responsible financial behavior—may be better able to manage financial stress and avoid material hardships (Sun & Chen, 2022), increase health care investment such as acquiring health products and services (Ling et al., 2023), and adopt healthy behaviors such as less smoking (Khan et al., 2021) or more frequent exercise (Yuktadatta et al., 2021). Sun and Chen’s (2022) study confirms the links between financial capability and health, showing that financial capability has a positive and longitudinal effect on health, independent of other critical social determinants such as race/ethnicity, gender, and socioeconomic status.

### Financial Capability and Health

Empirical studies examining financial capability have focused on the impacts of financial literacy and behavior on financial outcomes. A large body of literature supports the positive association between financial literacy (e.g., numeracy, computation, or knowledge) and behavior (e.g., money management, responsible spending, saving, borrowing, and planning behaviors) and financial well-being (Adam et al., 2017) and financial satisfaction (Friedline & West, 2016; West & Friedline, 2016; Xiao & O’Neill, 2018). These findings are largely supported by reviews and meta-analyses (Goyal & Kumar, 2021; Santini et al., 2019).

However, limited research has explored how financial capability affects physical or mental health. For example, Bennett et al. (2012) examined how financial and health literacy affect health-promoting behaviors and health status among older Americans. They found that financial literacy was negatively associated with lower levels of mobility disabilities, limitations in instrumental activities of daily living, depressive symptoms, and loneliness. Similarly, Kadoya et al. (2018) found a negative association between financial literacy and anxiety among Americans aged 40 and older. Research also shows that financial behavior predicts mental health outcomes. For instance, Taylor et al. (2011) report that those with higher financial capability—conceptualized as saving and money management behaviors—show lower psychological distress (measured by The 12-Item General Health Questionnaire [GHQ-12]). Weida et al. (2020) found an association between financial health, conceptualized as spending, saving, borrowing, and planning, and self-rated health and depressive symptoms. Lastly, Sun and Chen (2022) examined financial capability, measured as a single latent factor, including financial literacy, access, and behavior, and found that financial capability predicts general health status 4 years later.

Overall, the evidence shows that financial capability is a social determinant of health that can be addressed and intervened. However, several research gaps exist. First, empirical studies generally lack theoretical discussion on the conceptual link between financial capability and health. Second, previous studies conceptualize financial capability more as individual knowledge and behavior than the combination of individual ability and structural opportunity. There is one exception: Sun and Chen (2022) measured all aspects of financial capability with one latent construct, but such an approach is unable to find the most effective malleable intervention lever. Finally, the mechanisms and pathways between financial capability and health, especially the moderating role of gender, have not been extensively explored.

### Gender Variations

Under the framework of social determinants of health, gender, like other fundamental causes such as race and socioeconomic positions, reflects access to resources, power, and privileges (Link & Phelan, 1995). For example, research shows that women have fewer socioeconomic resources, as is evident in wage and wealth gaps (Chang, 2010). Financial capability, a precursor of financial resources and well-being, also shows significant gender differences. A growing body of literature identifies gender gaps in financial capability (Okamoto & Komamura, 2021; Robson & Peetz, 2020). For example, empirical research shows that women tend to have more positive financial behaviors and attitudes than men, but men tend to outperform women in financial knowledge (Kadoya & Khan, 2020). Significant differences are widely observed in financial literacy, with men tending to be more financially literate than women. For instance, the 2015 National Financial Capability Study among adults aged 18 and older showed that men scored 7.8% and 8% more than women in the sum scores of objective financial literacy tests (Al-Bahrani et al., 2019). Another U.S. study provides evidence of gender differences in financial literacy among older adults. Using a sample of 1,332 respondents aged 55+ from the 2008 Health and Retirement Study, Lusardi et al. (2009) found a stark gender gap in financial sophistication. Women were less knowledgeable about some complex financial and investment concepts, such as the working of the stock market, risk diversification, and asset pricing.

Research combining the evidence on the links between financial capability and health and gender differences in financial capability identifies the strong effects of gender on financial capability; such differences could be further translated into health. As Sun and Chen (2022) suggest, due to the structural and historical contexts, heterogeneous effects likely exist in the relationship between financial capability and health. Therefore, exploring how gender moderates the links between financial capability and health would promote an understanding of the intersectionality of gender, financial capability, and health.

### This Study

This study extends the literature in several ways. First, it looks beyond the financial outcomes of financial capability to focus on a comprehensive set of health and well-being characteristics. Second, it tests systematic components of the financial capability framework and examines the relative effect. Lastly, it explores the moderating effects of gender on the relationship between financial capability and health.

## Method

### Data and Sample

An online, city-wide survey was conducted between November 2020 and April 2021 to investigate healthy aging factors in Hong Kong among people aged 45 and older. The survey collected data on physical and mental health, demographics, lifestyle, and social and community activities. Individuals who met the age criteria, could read Chinese, and lived in Hong Kong were eligible to participate. A nonprofit organization invited respondents via a unique Google Forms link, where all responses were anonymous and informed consent was obtained before participation. Respondents who completed the survey were offered supermarket coupons sent via email or text messages; the contact information was deleted after the coupons were sent. A total of 1,109 people completed the survey. Ethics approval was obtained from Human Research Ethics Committee, The University of Hong Kong.

### Measures

#### Financial capability

Three aspects of financial capability (access, literacy, and behavior) were measured. Following Sun et al. (2022), *financial access* was assessed by eight binary measures of financial products and ownership (e.g., savings accounts, mortgages, life insurance, private medical insurance, annuities, stocks, derivatives, and foreign currency accounts). A higher score (*range*: 0–8) indicated better access to financial products and services. *Financial literacy* was measured by a one-item self-appraisal question to assess overall financial knowledge and understanding (*range*: 1–10, see Xiao & O’Neill, 2018); a higher score indicated a higher perceived literacy level. Lastly, *financial behavior* was evaluated using Lown’s (2011) financial self-efficacy scale, rated on a 4-point scale (1 = *exactly true*; 4 = *not at all true*). Three items regarding the use of credit, navigating financial challenges, and financial management were selected through exploratory factor analysis (Cronbach’s *α* = 0.90). This approach was similar to that of Rothwell et al. (2016) to identify a parsimonious measure for financial behavior. A higher score indicated positive financial behavior.

#### Health outcomes

Both positive and negative domains of physical, mental, and financial health were evaluated. *Physical health* was measured by two variables: self-rated health and mobility. Self-rated health was measured on a 10-point scale (1 = *poor*; 10 = *excellent*) in which participants rated their overall health, with higher scores indicating better health. Mobility limitation was originally rated on a 5-point scale based on the ability to perform basic physical tasks (e.g., walking). Due to high skewness, the variable was dichotomized into a binary variable indicating the presence of mobility limitations. *Mental health* was assessed by life satisfaction and depressive symptoms. Life satisfaction was measured on a 10-point scale (1 = *poor*; 10 = *excellent*), where participants rated their satisfaction levels. Depressive symptoms were evaluated using the 4-point (0 = *rarely*; 3 = *most of the time*), 10-item Center for Epidemiological Studies Depression scale, which has satisfactory reliability and validity in older Chinese populations (Chen & Mui, 2014). Two positive items (*happy* and *hopeful*) were reverse coded and summed with the remaining items; higher scores (*range*: 0–30) indicated more symptoms of depression (Cronbach’s *α* = 0.87). *Financial health* was evaluated by financial satisfaction and retirement worry. Financial satisfaction was assessed using the 4-point (0 = *very dissatisfied*; 4 = *very satisfied*), 6-item Satisfaction of Financial Situation scale (Hira & Mugenda, 1998), which rated participants’ satisfaction with six financial areas: savings, debt level, the family’s current financial situation, meeting long-term financial goals, dealing with financial emergencies, and money management skills. This measure had high internal reliability (Cronbach’s *α* = 0.91); higher scores indicated greater satisfaction with overall financial situations. Retirement worry was assessed on a 10-point scale (1 = *not worried*; 10 = *very worried*) measuring participants’ concern about their future retirement. We selected our health measures based on previous research by Bennett et al. (2012) for physical and mental health and Adam et al. (2017) for financial health, although these studies focused on financial literacy rather than a more comprehensive measure of financial capability.

#### Covariates

Following Santini and colleague’s (2019) systematic review, the covariates that correlated with financial capability and health were controlled for in the analyses: age, gender, marital status, whether having a college degree, whether having children, whether employed, number of chronic conditions, income, and assets. Table 1 documents the detailed categories for the covariates.

**Table 1.** Sample Characteristics and Gender Differences

Variables	All	Men	Women	Gender Diff.
	*M* (*SD*) or *N* (%)	*M* (*SD*) or *N* (%)	*M* (*SD*) or *N* (%)	*t*/*χ*^2^
Physical health				
Self-rated health	6.78 (1.93)	6.64 (1.97)	6.87 (1.91)	*t* = −1.88
Mobility limitation	235 (21.19%)	92 (21.10%)	143 (21.25%)	*χ* ^2^ = 0.003
Mental health				
Life satisfaction	6.94 (2.07)	6.77 (2.06)	7.05 (2.07)	*t *= −2.22*
Depressive symptoms	9.28 (5.72)	9.90 (5.78)	8.87 (5.65)	*t *= 2.92**
Financial health				
Worry about retirement	5.27 (2.63)	5.30 (2.60)	5.25 (2.66)	*t* = 0.30
Financial satisfaction	12.56 (4.48)	12.49 (4.63)	12.62 (4.38)	*t* = −0.47
Financial capability				
Financial access	2.39 (1.73)	2.13 (1.75)	2.56 (1.69)	*t* = −4.07***
Financial literacy	5.75 (2.09)	6.03 (2.06)	5.58 (2.09)	*t* = 3.51***
Financial behavior	6.52 (2.44)	6.44 (2.50)	6.57 (2.39)	*t* = −0.89
Covariates				
Age				
45–55	328 (29.58%)	104 (23.85%)	224 (33.28%)	*χ* ^2^ = 16.36***
56–64	521 (46.98%)	207 (47.48%)	314 (46.66%)	
65+	260 (23.44%)	125 (28.67%)	135 (20.06%)	
Marital status				
Married	672 (60.60%)	309 (70.87%)	363 (53.94%)	*χ* ^2^ = 32.67***
Single	287 (25.88%)	88 (20.18%)	199 (29.57%)	
Divorced or widowed	150 (15.53%)	39 (8.94%)	111 (16.49%)	
Having children	655 (59.06%)	284 (65.14%)	371 (55.13%)	*χ* ^2^ = 10.69***
Having college degree	638 (57.53%)	255 (58.49%)	383 (56.91%)	*χ* ^2^ = 0.26
In employment	495 (44.63%)	193 (44.27%)	302 (44.87%)	*χ* ^2^ = 0.03
Number of chronic illnesses	0.74 (0.92)	0.92 (0.97)	0.63 (0.87)	*t* = 5.11***
Monthly income				
No income	346 (32.20%)	138 (31.65%)	208 (30.91%)	*χ* ^2^ = 12.11*
<10K HKD	219 (19.75%)	66 (15.14%)	153 (22.73%)	
10–30K HKD	294 (26.51%)	119 (27.29%)	175 (26.00%)	
30–50K HKD	118 (10.64%)	56 (12.84%)	62 (9.21%)	
50–70K HKD	54 (4.87%)	24 (5.50%)	30 (4.46%)	
70K+	78 (7.03%)	33 (7.57%)	45 (6.69%)	
Assets				
No assets	147 (13.55%)	74 (17.09%)	73 (11.20%)	*χ* ^2^ = 11.27
<0.5 million HKD	457 (42.12%)	167 (38.57%)	290 (44.48%)	
0.5–1 million HKD	204 (18.80%)	79 (18.24%)	125 (19.17%)	
1–5 million HKD	89 (8.20%)	42 (9.70%)	47 (7.21%)	
5 million + HKD	188 (17.33%)	71 (16.40%)	117 (17.94%)	

*Note*: *p < .05. **p < .01. ***p < .001.

### Statistical Analyses

Descriptive and bivariate analyses (*t* and *χ*^2^ tests) were used to describe and compare sample characteristics. We used hierarchical linear and logistic regression models to examine the moderating effect of gender on financial capability (i.e., literacy, access, and behavior) and physical, mental, and financial health. We examined the components of financial capability individually, followed by testing all three financial capability measures simultaneously to explore the relative effects of financial capability on health. We used mean-centering for the financial capability measures and included the product terms of gender with financial access, literacy, and behavior to investigate the moderated effects. All of these models were controlled for the covariates. We visualized the moderating effect by plotting the simple slopes of financial capability by gender (Aiken et al., 1991). The largest variance inflation factor value was 4.48, indicating no severe multicollinearity issues. A nonsignificant Little’s test (*χ*^2^ = 10.54, *p* = .48) indicated that the missing cases were completely at random. Therefore, listwise deletion was used in the analyses.

## Results

### Sample Characteristics

Table 1 presents the overall sample characteristics and gender differences. Approximately 70% were aged 56 and older. Approximately 60% were married, had children, or had a college degree. Approximately 40% of the respondents were in employment. One-third reported having no income, and 13% reported having no assets. Significant gender differences were observed in age, marital status, income, and assets. Women were younger (*χ*^2^ = 16.36, *p* < .001), more likely to be single or divorced (*χ*^2^ = 32.67, *p* < .001), and had lower incomes (*χ*^2^ = 12.11, *p* < .05). Although no differences were found in physical and financial health, significant gender differences in financial capability and mental health were observed. Compared to men, women had greater financial access (*t* = −4.07, *p* < .001) but lower financial literacy (*t* = 3.51, *p* < .001); women also had higher life satisfaction scores (*t* = −2.22, *p* < .05) and lower depressive symptoms (*t* = 2.92, *p* < .01). The correlation results (see Supplementary Table 1) showed that financial capability was significantly associated with health. Both financial literacy and behavior had a stronger correlation with self-rated health (*r* = 0.46–0.60, *p* < .05), life satisfaction (*r* = 0.46–0.64, *p* < .05), and financial satisfaction (*r* = 0.50–0.80, *p* < .05), but showed a weak association with mobility (*r* = −0.07 to 0.27, *p* < .05) and depressive symptoms (*r* = −0.14 to 0.29, *p* < .05).

### Multivariate Analyses

**Figure 1.**
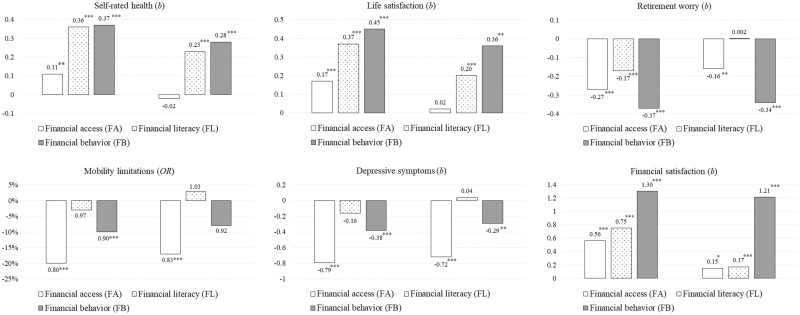
Independent and relative importance of financial capability components on health before (left panel) and after (right panel) adjusting for financial capability. *Note*: All models were adjusted for covariates in Table 1. *p < .05, **p < .01, ***p < .001.

Table 2 and Figure 1 present the linear and logistic regression results of financial capability on three health domains and interaction effects by gender. The left panel of Figure 1 shows the *independent* effect of financial literacy, access, and behavior on health outcomes when each financial capability variable was modeled as a single predictor, whereas the right panel of Figure 1 shows the *relative* effect of financial capability when all three variables were modeled simultaneously. As shown in the left panel of Figure 1, except for the associations between financial literacy and mobility limitations and depressive symptoms, all components of financial capability were related to physical, mental, and financial health when other financial capability factors were not controlled in the models. However, when the models controlled for other financial capability factors (Table 2 and the right panel of Figure 1), each aspect of financial capability demonstrated distinct effects on varied health outcomes. For example, financial access was significantly associated with lower mobility limitations (OR = 0.83, *p* < .01), depressive symptoms (*b* = −0.72, *p* < .001), and worry about retirement (*b* = −0.16, *p* < .001). Financial literacy was positively correlated with self-rated health (*b* = 0.23, *p* < .001), life satisfaction (*b* = 0.20, *p* < .001), and financial satisfaction (*b* = 0.17, *p* < .001). Lastly, there was a positive correlation between financial behavior and three aspects of health. Those with positive financial behavior reported higher levels of self-rated health (*b* = 0.28, *p* < .001), life satisfaction (*b* = 0.36, *p* < .001), and financial satisfaction (*b* = 1.21, *p* < .001), but lower levels of worry for retirement (*b* = −0.34, *p* < .001) and depressive symptoms (*b* = −0.29, *p* < .01). Overall, financial access and financial behavior, compared to financial literacy, showed a relatively stronger prediction to health across all domains.

**Table 2.** Moderated Effect of Gender on Financial Capability and Health Outcomes

Variables	Physical health				Mental health				Financial health			
	Self-rated health		Mobility limitation		Life satisfaction		Depressive symptoms		Retirement worry		Financial satisfaction	
	*b* (*SE*)	*b* (*SE*)	*OR*	*OR*	*b* (*SE*)	*b* (*SE*)	*b* (*SE*)	*b* (*SE*)	*b* (*SE*)	*b* (*SE*)	*b* (*SE*)	*b* (*SE*)
Financial capability												
Financial access (FA)	−0.02 (0.03)	−0.02 (0.05)	0.83**	0.77*	0.02 (0.03)	0.09 (0.05)	−0.72*** (0.12)	−0.69*** (0.18)	−0.16*** (0.05)	−0.05 (0.07)	0.15* (0.06)	0.14 (0.09)
Financial literacy (FL)	0.23*** (0.03)	0.34*** (0.04)	1.03	0.92	0.20*** (0.03)	0.29*** (0.04)	0.04 (0.09)	−0.11 (0.15)	0.002 (0.04)	−0.10 (0.06)	0.17*** (0.05)	0.12 (0.08)
Financial behavior (FB)	0.28*** (0.03)	0.22*** (0.04)	0.92	0.92	0.36*** (0.03)	0.24*** (0.04)	−0.29** (0.09)	−0.20 (0.14)	−0.34*** (0.04)	−0.37*** (0.06)	1.21*** (0.05)	1.24*** (0.07)
Gender interaction												
FA × gender		0.001 (0.06)		1.09		−0.11^†^ (0.06)		−0.05 (0.22)		−0.18* (0.09)		0.01 (0.11)
FL × gender		−0.18** (0.05)		1.19		−0.16** (0.06)		0.25 (0.19)		0.16^†^ (0.08)		0.09 (0.09)
FB × gender		0.11* (0.05)		0.98		0.21*** (0.05)		−0.16 (0.17)		0.05 (0.07)		−0.06 (0.09)
Model fits												
* R* ^2^	0.46	0.47	0.16	0.17	0.47	0.48	0.17	0.17	0.31	0.32	0.66	0.66
* R* ^2^ change		0.01**		0.01		0.01***		0.00		0.01*		0.00

*Note*: Models were controlled for age, gender, marital status, having children, education level, work status, number of chronic conditions, income, and assets. *R*^2^ for mobility limitation was pseudo *R*^2^.

^†^ *p* < .10. *p < .05. **p < .01. ***p < .001.

**Figure 2.**
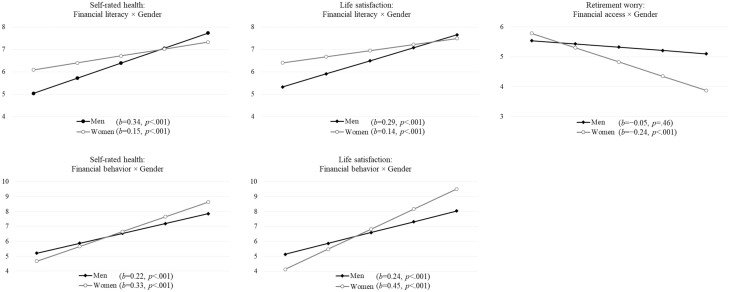
Moderated effect of gender between financial capability components and health. *Note*: The simple slope analyses for interaction terms with a *p* value less than .10 were not plotted as the simple slopes were insignificant.

Lastly, significant gender differences in financial capability were observed in self-rated health, life satisfaction, and retirement worry (see Table 2). However, the effects of each aspect of financial capability on health outcomes varied between men and women. As Figure 2 shows, financial literacy had a stronger impact on health among men, but the effects of financial behavior were apparent among women. Men who had higher levels of financial literacy had better self-rated health (*b* = 0.34, *p* < .001) and life satisfaction (*b* = 0.29, *p* < .001). In contrast, women who had positive financial behavior had high levels of self-rated health (*b* = 0.33, *p* < .001) and life satisfaction (*b* = 0.45, *p* < .001). Furthermore, financial access was negatively associated with retirement worry, but such an effect was only significant in women (*b* = −0.24, *p* < .001). No gender differences were found in mobility limitations, depressive symptoms, and financial satisfaction.

## Discussion

Despite increasing interest in promoting financial capability to enhance desirable outcomes, few studies have examined whether financial capability, including literacy, access, and behavior, relates to health. This study examined three critical questions: Does financial capability affect health? If so, what particular aspects of financial capability are most influential? Additionally, are there gender differences in the associations between financial capability and health? We found that financial capability is associated with health overall, but the effects of each aspect of financial capability vary depending on the specific health outcome. Furthermore, significant gender differences are observed in the links between financial capability and health, suggesting that gender-specific pathways to financial capability may lead to health disparities.

### Financial and Health Impacts of Financial Capability

Our study found that financial capability is significantly associated with financial outcomes. Consistent with previous research (Friedline & West, 2016; West & Friedline, 2016; Xiao & O’Neill, 2018), financial access, literacy, and behavior were associated with higher financial satisfaction. Moreover, our study showed that financial access and behavior, but not financial literacy, were associated with lower levels of retirement worry. This suggests that being financially inclusive and responsible, rather than simply being financially literate or confident, could effectively reduce retirement worry. Previous research has indicated that, compared to financial literacy, having access to mainstream financial products and services (such as savings accounts or credit services; see Despard et al., 2020; Friedline & West, 2016) can inform responsible financial planning, including short-term bill payment, medium-term emergency fund savings, and long-term retirement savings (Nam & Loibl, 2021). Our findings imply that improving financial literacy is necessary to strengthen financial well-being and retirement security but may not be as effective as increasing financial inclusion and remove barriers to accessing financial products and services (Sun et al., 2022).

This study expands the existing literature regarding the relationship between financial capability and health outcomes. Specifically, our research goes beyond the scope of financial literacy (e.g., Bennett et al., 2012; Taylor et al., 2011) and examines the role of financial access and behavior concerning both physical and mental health. The findings indicate that individuals with higher levels of financial literacy and positive financial behavior report better self-rated health and overall life satisfaction. Conversely, those with better financial access and behavior report experiencing fewer mobility limitations and symptoms of depression. Previous studies have found that financial capability facilitates better health through mental and physical mechanisms. Financial capability is associated with better planning and coping behaviors, which can lead to lower levels of financial stress and greater financial stability (Kadoya et al., 2018). As a result, individuals with higher financial capability are likely to experience lower rates of anxiety, psychological distress, and other stress-related illnesses. Furthermore, these individuals are more likely to have the necessary resources to afford a healthy diet, exercise regularly, and make other lifestyle choices that contribute to good health (Sun & Chen, 2022).

Although each aspect of financial capability is associated with varied health outcomes, our study shows they demonstrate varying relative importance on health. Among the three aspects of financial capability, financial behavior is considered a stronger health predictor than financial literacy and access. Financial capability theory hypothesizes that individuals with sound financial capability enjoy higher financial literacy, better financial access, and positive financial behavior. However, financial knowledge and the availability of affordable, safe, mainstream financial products and services can also shape financial behavior (Sherraden, 2013). This study explored the relative impacts of financial capability and the gendered mechanism on health and did not aim to explore this potential mediation. However, such a theoretical proposition implies that financial behavior is more of an immediate downstream determinant of health, possibly explaining the consistent associations between financial behavior and health outcomes.

### Gendered Pathways to Financial Capability and Health

Gender significantly affects an individual’s financial capability (Preston & Wright, 2019; Robson & Peetz, 2020), which in turn may lead to health disparities between men and women. Our study confirms these assumptions, as financial access and literacy varied significantly by gender, despite the bivariate analyses revealing no gender differences in health. Multivariate analyses revealed that gender moderated the relationships between financial capability and health, particularly for self-rated health, life satisfaction, and retirement worry. Financial literacy had a stronger impact on men’s self-rated health and life satisfaction, whereas financial behavior was more evident among women. The gender difference was also evident in retirement worry, where having financial access was not related to retirement worry among men, but a significant reduction in retirement worry was observed among women.

Gender differences in financial literacy have been linked to health disparities, with several possible explanations proposed in the literature. One such explanation is the socialization process, which may limit women’s exposure to financial matters. Stereotypical beliefs about women’s abilities in math and money management, and expectations about gender roles, may discourage women from learning about finances and undervalue financial issues (Tinghög et al., 2021). Over time, these early disadvantages may lead to a significant gap in financial literacy between men and women that persists into adulthood. For example, women are more likely to answer “don’t know” in financial tests, rendering them more likely to be classified as having low knowledge (Lusardi et al., 2009; Tinghög et al., 2021). Another possible factor is marital status, where men are more likely to be the primary decision-makers on saving, investment, and money management issues, particularly if their female partner is less educated. This can limit women’s exposure to financial development and leave them more financially vulnerable (Fonseca et al., 2012; Preston & Wright, 2019). These factors may explain why the impacts of financial literacy on men are stronger than on women when examining the associations between financial literacy and health.

Compared to men, women’s financial access and behavior are more important when assessing the impact of financial capability on perceived health and retirement security. This gender difference may reflect variations in life course trajectories and occupational pathways that affect the ability to build savings and retirement plans. Women are more likely to face financially disadvantageous situations due to disruptive events in their working lives, such as caregiving or marriage, leading to them taking on a disproportionate share of unpaid household and care work. Consequently, women tend to work fewer hours in paid employment and earn less income over their lifetime, limiting their propensity to plan and their opportunity for financial development (Preston & Wright, 2019). In this sense, the constraints of women’s life experiences limit their propensity to plan and opportunities for financial development. Therefore, women may be more conscious and perceive the need for future savings, motivating them to be financially prudent, savings-oriented, and risk-averse (Kadoya & Khan, 2020). We conducted sensitivity tests comparing gender differences in financial access to test this assumption. The results (see Supplementary Table 2) showed that women are more likely to own financial products that would be regarded as conservative, such as bank deposits, life insurance, private medical insurance, and annuities. However, there were no gender differences in ownership of stocks, derivatives, or foreign currency. This explains why women have a higher financial access score than men in our study and that financial behavior has a much stronger effect on women.

### Limitations

The study’s findings should be considered in the light of several limitations. First, although theoretical and empirical evidence suggests that financial capability is a health determinant (Sun & Chen, 2022), the findings indicate association rather than causation due to the cross-sectional design. Second, online sampling may be prone to selecting financially savvy respondents, potentially influencing the findings’ generalizability. Third, although research suggests that subjective financial literacy is as valuable as objective financial literacy (Allgood & Walstad, 2016), the lack of objective financial literacy in this study may result in a partial understanding of the impacts of financial literacy on health. Fourth, using a sample aged 45 and older may raise concerns regarding the associations between financial capability and health. Although age was adjusted as a covariate, previous research has indicated that age differences exist in financial capability (Xiao et al., 2015), which may subsequently affect health outcomes. A sensitivity test of bivariate analyses of financial capability and health by age (see Supplementary Table 3) demonstrated that older adults (aged 65+) reported lower self-rated health, more mobility limitations, less retirement worry, and lower financial access compared to those aged 45–64. However, the results of multivariate analyses (see Supplementary Table 4) provide little support for the moderating effect of age, suggesting that the effects of financial capability on health were similar for both middle-aged and older adults. Fifth, similar to prior research examining financial capability in highly developed or urban contexts such as the United States, UK, or Japan, our findings—based on Hong Kong as a highly developed international city—may not be generalizable to developing areas in which the opportunities and ability to receive financial education, use financial services, and build positive financial behaviors may be limited. Future research should explore how financial capability may operate in developing regions to examine its role in health. Finally, the gender construct was limited to binary categorical measurement, although gender is complex and multidimensional. Research has found that masculinity is associated with better self-rated health for cisgender men, whereas femininity is associated with better health for cisgender women (Hart et al., 2019). Our sample was limited to cisgender adults; information on transgender, nonbinary, and gender nonconforming identities was not available in this study.

## Implications and Conclusion

This study has implications for program design, policy, and research. By comprehensively examining financial literacy, access, and behavior and their impacts on health, this study shows that financial capability not only lies in an individual’s knowledge and behavior but also in access to financial services, products, and policies. Therefore, improving financial capability requires multidimensional interventions. A policy or program that only targets one aspect of financial capability without a holistic action is an oversimplification of financial capability and may produce futile interventions in further improving health. Furthermore, this study provides empirical evidence that financial capability is a determinant associated with a wide range of health outcomes. The findings reveal that addressing financial capability can potentially improve health and well-being. The significant associations between financial capability and health suggest that financial health is integral to overall health. Understanding and addressing an individual’s financial health is as important as caring for their physical and mental health (Weida et al., 2020). Thus, financial and health-integrated services should be offered in social and health settings. Examples include offering credit counseling and financial guidance in social services and health clinics or assessing clients’ financial profiles (e.g., situations, hardships, or stress) and health and psychosocial assessments during intake (Sun et al., 2022; Weida et al., 2020).

This study also demonstrates that gender moderates the relationship between financial capability and health. Due to patriarchy and gendered socialization and expectations, gender differences that occurred early in financial capability could be later translated into health disparities between men and women. Considering that women may experience more life events with the potential to disrupt the continuity of their working lives and financial development, the policy should be inclusive by accounting for these life course challenges. Policy or programs, such as inclusive retirement accounts, promotion of financial access, and provisions of tailored financial guidance, particularly for women, may enhance financial capability and health in later life. Lastly, future studies should examine further the financial capability-health nexus. This study shows that financial capability is a social determinant of health. As Braveman and Gottlieb (2014) suggested, the relationships between social determinants and health are complex, dynamic, and interactive, which may involve multiple mechanisms, though these mechanisms through which financial capability may affect health are underexplored. For example, financial capability may enhance knowledge, literacy, or coping behaviors associated with increased financial resources, thereby affecting health outcomes through positive lifestyle behaviors (e.g., less smoking, frequent exercise, or a healthy diet). Nevertheless, the links between financial capability and health could be captured by increased income associated with better housing conditions, neighborhood environment, or lower stress. Lastly, the associations could be mediated by control belief, social standing (i.e., perceived social status), or social network, which may strengthen individuals’ problem-solving capacity, social and economic resources, social support, and norms for healthy social behaviors. As there are long time lags for health effects to manifest through financial capability, research that employs longitudinal or experimental design or investigates the dynamics or mediational pathways via socioeconomic and biopsychological characteristics should be promoted to investigate the nuanced links between financial capability and health.

In conclusion, this study confirms the positive associations between financial capability and a constellation of health outcomes, with significant gender differences identified in these relationships. The findings suggest that expanding financial access and improving individuals’ financial knowledge and behaviors may improve health and well-being in later life. Such policy and programming efforts should also consider gender and context-specific pathways to promote financial capability to reduce economic and health inequalities in later life.

## Supplementary Material

igad072_suppl_Supplementary_Material
