# Supplementary material for: Gender Differences in the Relationship Between Financial Capability and Health in Later Life: Evidence From Hong Kong
Source: Innov Aging. 2023 Jul 7;7(6):igad072. doi: 10.1093/geroni/igad072 (PMC10406417; doi:10.1093/geroni/igad072)
Supplement: igad072_suppl_Supplementary_Material [file igad072_suppl_supplementary_material.docx]

**Online Supplementary Materials**

**Table S1.** Correlation of Financial Capability and Health Outcomes

| Variables | 1 | 2 | 3 | 4 | 5 | 6 | 7 | 8 | 9 |
| --- | --- | --- | --- | --- | --- | --- | --- | --- | --- |
| 1. Financial access | -- | 0.35* | 0.48* | 0.32* | −0.19* | 0.34* | −0.33* | −0.35* | 0.46* |
| 2. Financial literacy | 0.35* | -- | 0.58* | 0.46* | −0.07 | 0.46* | −0.14* | −0.21* | 0.53* |
| 3. Financial behavior | 0.48* | 0.56* | -- | 0.57* | −0.18* | 0.64* | −0.29* | −0.41* | 0.78* |
| 4. Self-rated health | 0.37* | 0.60* | 0.60* | -- | −0.29* | 0.77* | −0.31* | −0.31* | 0.55* |
| 5. Mobility limitation | −0.28* | −0.22* | −0.27* | −0.37* | -- | 0.18* | 0.23* | 0.17* | −0.18* |
| 6. Life satisfaction | 0.43* | 0.56* | 0.60* | 0.78* | −0.28* | -- | −0.33* | −0.37* | 0.61* |
| 7. Depressive symptoms | −0.33* | −0.20* | −0.29* | −0.31* | 0.23* | −0.35* | -- | 0.48* | −0.33* |
| 8. Worry about retirement | −0.31* | −0.33* | −0.51* | −0.34* | 0.15* | −0.44* | 0.49* | -- | −0.50* |
| 9. Financial satisfaction | 0.46* | 0.50* | 0.80* | 0.60* | −0.28* | 0.63* | −0.31* | −0.54* | -- |

*Note.* The lower triangle indicates correlations for men and the upper triangle correlations for women. **p* < .05

**Table S2.** Bivariate Analyses of Financial Access by Gender

| Financial access | Men (%) | Women (%) | *χ^2^* |
| --- | --- | --- | --- |
| Bank deposit | 65.14 | 78.16 | 22.78*** |
| Mortage | 8.94 | 10.25 | 0.51 |
| Life insurance | 34.17 | 40.56 | 4.58* |
| Private medical insurance | 20.41 | 33.58 | 22.56*** |
| Annuity | 19.27 | 24.81 | 4.65* |
| Stock | 42.89 | 42.2 | 0.05 |
| Derivatives | 7.34 | 7.73 | 0.06 |
| Foreign currency | 15.60 | 19.47 | 2.69 |

**p* < .05, ***p* < .01, ****p* < .001

**Table S3.** Bivariate Analyses of Health Outcomes and Financial Capability by Age

| Variables | Aged 45-64 | Aged 65+ | *t or χ^2^* |
| --- | --- | --- | --- |
|  | *M*(*SD*) or *N*(%) | *M*(*SD*) or *N*(%) |  |
| Physical health |  |  |  |
| Self-rated health | 6.87 (1.89) | 6.48 (2.03) | *t* = 2.83** |
| Mobility limitation | 136 (16.02%) | 99 (38.08%) | *χ^2^* = 57.99*** |
| Mental health |  |  |  |
| Life satisfaction | 6.96 (2.04) | 6.88 (2.15) | *t* = 0.54 |
| Depressive symptoms | 9.39 (5.68) | 8.90 (5.86) | *t* = 1.20 |
| Financial health |  |  |  |
| Worry about retirement | 5.43 (2.64) | 4.74 (2.53) | *t* = 3.74** |
| Financial satisfaction | 12.56 (4.42) | 12.57 (4.66) | *t* = −0.01 |
| Financial capability |  |  |  |
| Financial access | 2.51 (1.74) | 2.01 (1.63) | *t* = 4.10*** |
| Financial literacy | 5.81 (2.12) | 5.69 (1.98) | *t* = 1.67 |
| Financial behavior | 6.56 (2.41) | 6.40 (2.52) | *t* = 0.92 |

**p* < .05, ***p* < .01, ****p* < .001

**Table S4.** Moderated Effect of Age on Financial Capability and Health Outcomes

| Variables | Physical well-being | | Mental well-being | | Financial well-being | |
| --- | --- | --- | --- | --- | --- | --- |
|  | Self-rated  health | Mobility  limitation | Life  satisfaction | Depressive  symptoms | Retirement  worry | Financial  satisfaction |
|  | *b (SE)* | *OR* | *b (SE)* | *b (SE)* | *b (SE)* | *b (SE)* |
| Financial capability |  |  |  |  |  |  |
| Financial access (FA) | −0.03  (0.03) | 0.84*** | 0.01  (0.03) | −0.66***  (0.13) | −0.14**  (0.05) | 0.13*  (0.06) |
| Financial literacy (FL) | 0.21***  (0.02) | 1.02 | 0.18***  (0.03) | 0.14  (0.10) | −0.02  (0.04) | 0.21***  (0.05) |
| Financial behavior (FB) | 0.28***  (0.02) | 0.90 | 0.36***  (0.02) | −0.40***  (0.10) | −0.39***  (0.04) | 1.18*** |
| Age interaction |  |  |  |  |  |  |
| FA × age | 0.06  (0.07) | 0.91 | 0.04  (0.07) | −0.34  (0.27) | −0.10  (0.11) | 0.12  (0.13) |
| FL × age | 0.06  (0.06) | 1.02 | 0.05  (0.06) | −0.38  (0.23) | 0.18†  (0.10) | −0.14  (0.11) |
| FB × age | −0.03  (0.05) | 1.02 | −0.02  (0.05) | 0.34†  (0.20) | 0.12  (0.09) | 0.11  (0.10) |

*Note.* Models were controlled for age, gender, marital status, having children, education level, work status, number of chronic conditions, income, and assets. ^†^*p* < .10, **p* < .05, ***p* < .01, ****p* < .001
